# Social network distribution of syphilis self-testing among men who have sex with men in China: study protocol for a cluster randomized control trial

**DOI:** 10.1186/s12879-021-06137-0

**Published:** 2021-05-27

**Authors:** Yajie Wang, Wei Zhang, Dongping Bao, Jason J. Ong, Joseph D. Tucker, Rouxuan Ye, Heping Zheng, Bin Yang, Cheng Wang

**Affiliations:** 1 Dermatology Hospital of Southern Medical University, Guangzhou, Guangdong China; 2 Southern Medical University Institute for Global Health and Sexually Transmitted Diseases, Guangzhou, Guangdong China; 3 Guangdong Provincial Center for Skin Disease and STI Control, Guangzhou, Guangdong China; 4 University of North Carolina at Chapel Hill, Project-China, Guangzhou, Guangdong China; 5 Faculty of Infectious and Tropical Diseases, London School of Hygiene and Tropical Medicine, London, UK; 6 Central Clinical School, Monash University, Melbourne, Victoria Australia; 7 Institute for Global Health and Infectious Diseases, School of Medicine, University of North Carolina at Chapel Hill, Chapel Hill, USA; 8 Department of Biostatistics, Southern Medical University, Guangzhou, Guangdong China

**Keywords:** Syphilis, Self-test, MSM, Social network distribution, cRCT

## Abstract

**Background:**

Syphilis is a common sexually transmitted infection (STI) among men who have sex with men (MSM). Increasing syphilis testing is important to syphilis control. However, in low- and middle-income countries like China, syphilis testing rates remain low among MSM. We describe a randomized controlled trial protocol to examine the effectiveness of social network distribution approaches of syphilis self-testing among MSM in China.

**Methods:**

We will recruit index and alter MSM. Indexes will be eligible if they: are born biologically male; aged 18 years or above; ever had sex with another man; are willing to distribute syphilis testing packages or referral links to their alters; and willing to provide personal contact information for future follow-up. Three hundred MSM will be recruited and randomly assigned in a 1:1:1 ratio into three arms: standard of care (control arm); standard syphilis self-testing (SST) delivery arm; and referral link SST delivery arm. Indexes will distribute SST packages or referral links to encourage alters to receive syphilis testing. All indexes will complete a baseline survey and a 3-month follow-up survey. Syphilis self-test results will be determined by photo verification via a digital platform. The primary outcome is the mean number of alters who returned verified syphilis testing results per index in each arm.

**Discussion:**

The trial findings will provide practical implications in strengthening syphilis self-testing distribution and increasing syphilis testing uptake among MSM in China. This study also empowers MSM community in expanding syphilis testing by using their own social network.

**Trial registration:**

Chinese Clinical Trial Registry, ChiCTR2000036988. Registered 26 August 2020 - Retrospectively registered.

**Supplementary Information:**

The online version contains supplementary material available at 10.1186/s12879-021-06137-0.

## Background

Syphilis remains a global health priority. The WHO estimated that the prevalence of syphilis among men who have sex with men (MSM) is 5% or more in at least 42 countries in 2018 [1]. From 2006 to 2012, the incidence of syphilis was 9.6 per 100 person-years among MSM living in China [2]. However, in low- and middle-income countries (LMICs) like China, syphilis testing rate is low among MSM. Studies showed that less than 30% of Chinese MSM has ever received a syphilis test [3].

Facility-based testing for syphilis has potential limitations related to stigma from providers [4], infrastructure requirement, inconvenience [5], and lack of privacy [6]. Recent advances in diagnostics such as self-testing, have enabled decentralized testing strategies [7] which makes testing more accessible. Syphilis self-testing is a process whereby an individual collects their own specimen, performs the test and interprets the result by themselves [3]. Several point-of-care syphilis tests have been approved and used for syphilis screening in China [8, 9]. A cross-sectional study showed that syphilis self-testing among MSM could complement facility-based testing in China [3]. Approximately half of the MSM reported that syphilis self-testing was their first syphilis test, suggesting that this could expand test uptake among groups without a history of testing [3]. A recent randomized control trial reported that syphilis self-testing significantly increased syphilis testing among Chinese MSM with less cost per person tested compared to facility-based testing [10]. With the potential benefits of syphilis self-testing, further studies are needed to explore how this could be scaled up.

Social network distribution can be an effective syphilis self-testing distribution strategy to expand syphilis testing and to reach high-risk populations with undiagnosed syphilis. This strategy allows participants (defined as indexes) to apply for multiple self-testing kits and distribute to their peers (sexual partners or non-sexual partners, defined as alters) within their social network [11, 12]. Social network distribution has been widely used for sexual partner notification with notification cards, which helps contain the spread of sexually transmitted infections (STIs) [13, 14]. However, this strategy is likely to reach only a proportion of contacts. For example, it is more successful in reaching partners in long-term relationships and less so in reaching short-term casual sexual contacts [14, 15]. Social network distribution of self-testing may be an effective approach to overcome these barriers [16]. A study showed that social network testing enables people at high risk for HIV or people living with HIV to encourage people in their social network to test for HIV [17]. This strategy is highly acceptable among MSM because it is distributed by trusted community leaders [18]. Social network distribution may reduce health disparities by decreasing barriers such as stigma and discrimination in health facilities [19], concerns for lack of confidentiality [20], and fear of sexuality disclosure [21] among hard-to-reach populations [17]. Given the feasibility of this strategy in expanding HIV self-testing among MSM, there is value in exploring whether this distribution strategy could also expand syphilis self-testing.

This study aims to examine the effectiveness of social network distribution approaches of syphilis self-testing through a three-arm cluster randomized controlled trial (cRCT) in Guangdong, China and to explore which approach could increase syphilis testing among Chinese MSM.

## Methods

### Study design

**Fig. 1:**
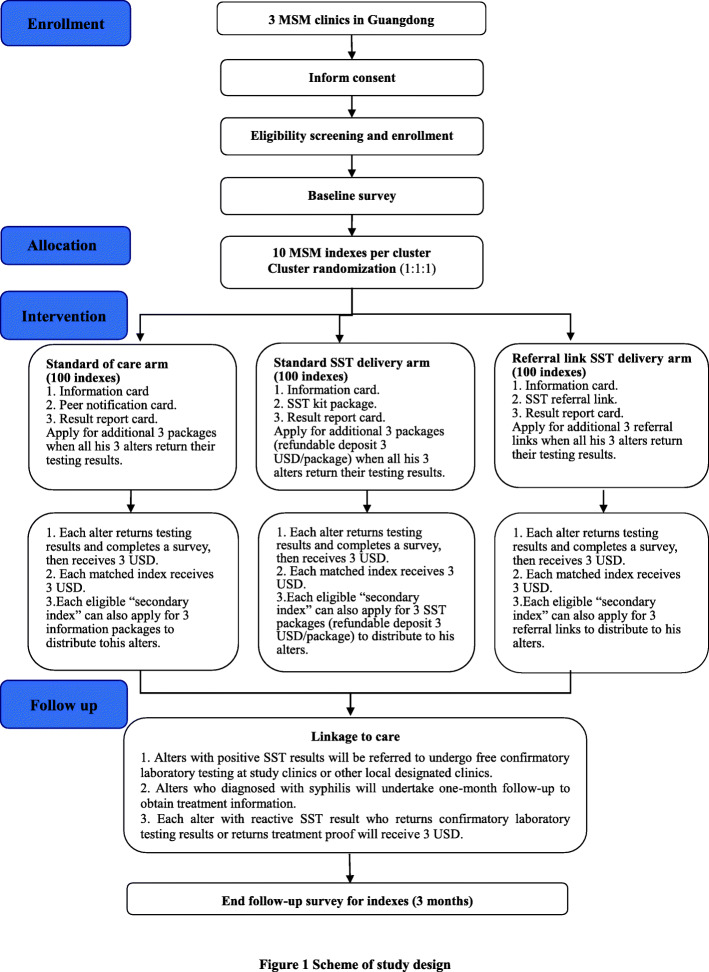
Scheme of study design

This is a non-blinded and parallel three-arm cluster randomized controlled trial among MSM living in China. Enrolled indexes will be randomly assigned in a 1:1:1 ratio into three arms: standard of care arm (control arm); standard SST delivery arm; and referral link SST delivery arm (referral link is used to apply for free SST packages). Indexes in the control arm will receive information packages with peer notification cards to encourage their alters to take a free syphilis screening at a designated health facility. Indexes in the standard SST delivery arm will receive free packages containing a SST kit to distribute to their alters. Indexes in the referral link SST delivery arm will be provided with SST referral links to distribute to their alters to access free syphilis self-testing packages online. Indexes in each arm will be followed up for 3 months (Fig. 1). The hypothesis in this study is that standard and referral link syphilis self-testing social network delivery model are more effective than standard of care among Chinese MSM. Preliminary data from pilot study will be used to inform the final trial design. Preliminary pilot results are available on the Supplement materials.

### Study setting and population

All indexes will be recruited by health workers at local MSM voluntary counselling and testing (VCT) clinics (in Foshan and Dongguan) or community-based organizations (CBOs) (in Shenzhen) from three cities in Guangdong Province, China. All sites are run by MSM community-based organizations (Xinghuo LGBT center, Shenzhen; Friends Care Center, Foshan; and Rainbow Center, Dongguan) and provide free HIV/STI screening and consultations for MSM. The staff at each site has rich experience with counselling, blood collection, rapid testing for syphilis, results reporting, and follow up of syphilis tests. All sites will follow the same study procedures.

Indexes will be eligible if they are: born biologically male; aged 18 years or above; ever had sex with another man; are willing to distribute syphilis testing packages or referral links to their alters; and willing to provide personal contact information for future follow-up. All participants need to sign an electronic informed consent before they fill out the baseline survey. Considering MSM living with HIV might be at higher risk of syphilis, we increase awareness of syphilis for MSM with HIV and mobilize health workers to include as many indexes with HIV as possible in the recruitment stage.

### Arms and interventions

Arms and interventions are summarized in Table 1.

**Table 1:** Group information

Arm	Intervention	Intervention delivery
**Standard of care arm**	1. **Information card**: the risk of acquiring syphilis, the importance of screening for syphilis, resources of three local syphilis test and linkage to care service sites, and a link of national syphilis VCT sites. 2. **Peer notification card**: social network distribution of notification card and encouragement of syphilis testing. 3. **Result report card**	1. Each index will receive 3 information packages at enrollment, and distribute packages to 3 alters. Maximum **ONE** package per alter. 2. Each alter who returns his testing result and completes a survey can receive 3 USD and his matched index will also receive an extra 3 USD. 3. Each index can apply for additional 3 packages when all his 3 alters return testing results. 4. Each eligible “secondary index” can apply for 3 information packages to distribute to his alters.
**Standard SST delivery arm**	1. **Information card**: the risk of acquiring syphilis, the importance of screening for syphilis, resources of three local syphilis test and linkage to care service sites, and a link of national syphilis VCT sites. 2. **SST kit package**: social network distribution of SST kit package and encouragement of SST. 3. **Result report card**	1. Each index will receive 3 SST packages at enrollment, and distribute packages to 3 alters. Maximum **ONE** package per alter. 2. Each alter who returns his testing result and completes a survey can receive 3 USD and his matched index will also receive an extra 3 USD. 3. Each index can apply for additional 3 packages (refundable deposit 3 USD/package) when all his 3 alters return testing results. 4. Each eligible “secondary index” can apply for 3 SST packages (refundable deposit 3 USD/package) to distribute to his alters.
**Referral link SST delivery arm**	1. **Information card**: the risk of acquiring syphilis, the importance of screening for syphilis, resources of three local syphilis test and linkage to care service sites, and a link of national syphilis VCT sites. 2. **SST referral link**: social network distribution of SST referral links and encouragement of SST. 3. **Result report card**	1. Each index will receive 3 SST referral links at enrollment, and distribute links to 3 alters. Maximum **ONE** link per alter. 2. Each alter can apply for **ONE** SST package through the referral link, and SST package will be posted to him free of charge. 3. Each alter who returns his testing result and completes a survey can receive 3 USD and his matched index will also receive an extra 3 USD. 4. Each index can apply for additional 3 referral links when all his 3 alters return testing results. 5. Each eligible “secondary index” can apply for 3 SST referral links to distribute to his alters.

#### Standard of care arm (control arm)

Each index MSM will initially receive three information packages to distribute to his alters after completing their baseline survey. Each information package contains: 1) a health promotion and linkage to care information card; 2) a crowdsourced peer notification card; and 3) a syphilis testing result report card. Crowdsourcing is a practice in which a group solves a problem and then shares the solutions with the community [22]. Peer notification contains healthcare provider location and index information, which each alter can use to receive a free syphilis screening in a list of nearby facilities for each study site. Each alter can use a maximum of one package and take one free facility-based syphilis testing service through peer notification card from each index.

#### Standard SST delivery arm

Index MSM in this arm will initially receive three SST packages to distribute to his alters after completing their baseline survey. Different from the information package for the control arm, we will give them SST kits instead of the peer notification card. Each SST kit contains equipment for blood sample collection, quick syphilis test and a step-by-step pictorial instruction for using the self-test kit. Each alter can use a maximum one SST package and take syphilis self-testing service from each index. In this trial, we will use the syphilis SD Bioline Syphilis 3.0 rapid test kit. The sensitivity and specificity for syphilis range 85.7–100% and 95.5–99.4% respectively [16, 23].

#### Referral link SST delivery arm

Index MSM in this arm will initially receive a unique SST referral link, which can be shared with up to three alters after baseline survey. Each link will expire after three times of usage. Each alter can only apply for one SST package with the link, which can be accessed by only one device (both a Wechat account and a phone number). Further, the SST package will be posted to the alter free of charge (free express delivery).

In all three study arms, each package or link will be assigned with a unique number for future returned results tracking and index matching. Alters will receive 3 USD when they send a photo verification of their self-testing or facility-testing results and complete the alter survey. Their matching index will also receive an extra 3 USD as incentive of successful distribution. In addition, each index can apply for additional three packages (refundable 3 USD deposit for each SST package; free of charge for notification card package) or for additional free referral links when their initial link has been used by three alters returning their test results. Further, each alter in all three arms who returned his result can become a “secondary index” if he is willing to and meets the following criteria: born biologically male, aged 18 years or above, and ever had sex with another man. He can also apply for three packages (refundable 3 USD deposit for each SST package; free of charge for information package) or referral link to distribute to his alters. For all indexes and alters in the study, researchers will provide a 24/7 hotline and online counselling.

### Randomization and allocation

At each site, eligible indexes will be assigned to one of the three arms through a cluster randomization procedure with ten participants per cluster. To ensure equal number of clusters in each of the three arms, the clusters will be randomized in a block of three at each study site. Computer-generated randomization codes will be produced and kept by a biostatistician who is not involved in participant enrollment. Ten blocks will be generated in total. Each block has three clusters consisting of standard of care arm, standard SST delivery arm, and referral link SST delivery arm, randomly created from all possible permutations. The schedule will be determined before the start of the study and then provided to each site. Each eligible index who presents to the site during a given 10-person cluster will be provided the allocated program. To reduce contamination between groups, eligible indexes will be allocated into the same group if they come in pair.

### Blinding

As it would not be possible to blind researchers and participants to participants’ group assignments, this is a non-blinded study.

### Follow up

The follow-up survey will be administered 3 months after recruitment for indexes. Each index will receive 4 USD after completing the follow-up survey. Any alters with a reactive self-testing results will be referred to undergo free confirmatory laboratory testing and clinical examination at study clinics or other local designated clinics/hospitals. If the participants are diagnosed with syphilis, we will undertake further follow-up to obtain treatment information. Alters with a reactive result who also return photo verification of confirmatory testing results or treatment proof will receive a further 3 USD. Alters with reactive results who do not return any proof for confirmation testing or treatment will be asked about further linkage to care information at the end of study.

### Outcome measures

#### Primary outcome

Mean number of alters who returned photo verified syphilis testing results per index in each arm, including facility-based test and self-testing in 3 months.

#### Secondary outcomes

1) Proportion of first-time syphilis testing among participants; 2) Proportion of testers with a positive syphilis testing result; 3) Economic evaluation in terms of total cost of implementing each of the three arms, the average cost per tester, the average cost per syphilis diagnosis, and the incremental cost-effectiveness ratio for the three arms; 4) Adverse events during the delivery procedures in each arm; 5) Mean number of alters who received the test kit; 6) Proportion of repeat testing among indexes and alters in each arm.

### Data collection

#### Syphilis testing record and results

All indexes’ information will be recorded using online surveys (Wenjuanxing online survey platform). Alters can photograph and upload their testing results to an online social media platform “WeChat” (instant messaging service/application) by scanning the QR code in the result report card. Syphilis testing results can be either from facility-based testing or self-testing.

### Surveys

#### Baseline survey for index

All eligible indexes will complete a baseline survey online on the web-based survey platform Wenjuanxing (Changsha Haoxing Information Technology Co., Ltd., China) at enrollment. The baseline survey collects information on socio-demographic information, history of sexual behaviors, syphilis testing, HIV testing and other STIs testing, and their social network. Sociodemographic information includes age, biological sex, residence status, marital status, highest education, monthly income, sexual orientation, and whether they disclosed their sexual orientation with others. Sexual behaviors include sexual history with men and/or women, role during sex with men, condom use, type of sex partners, group sex, and drug use. Facility-based testing or self-testing history of syphilis, HIV and other STIs, and treatment experience will also be collected. A set of questions including a scale to measure social support, community leadership, and the size of their social network will be asked [24, 25].

#### Alter survey

Each alter in the three arms will receive an online survey link after uploading their syphilis test results. The survey will be created on the Wenjuanxing platform and includes questions on the relationship between the indexes and the alters, sociodemographic information, sexual behavior, the experience of receiving syphilis testing packages or links, testing history of syphilis, HIV and other STIs, and social network. The experience of accepting syphilis testing packages or links includes the behavior of the indexes when delivering syphilis testing packages or links, and the satisfaction to the delivery process of syphilis testing packages or links. Adverse events will be asked to identify whether the alters were being forced to test, have received physical and/or verbal abuse, or being misunderstood from indexes etc. during the delivery procedures.

#### Follow-up survey

All indexes will complete a three-month follow-up survey online. The follow-up surveys will collect information of their experience of using the information packages, SST packages or referral link delivery; information on the relationship between the indexes and the alters; reasons of unwillingness to distribute; history of syphilis, HIV and other STIs testing in the past 3 months; and sexual behaviors in the past 3 months. Potential adverse events such as forced alters to test, received physical and/or verbal abuse, or being misunderstood from alters etc. during the delivery procures are asked.

### Cost data

A cost collection sheet will be used to identify, value and measure costs from the perspective of the program provider. This will include the start-up costs (e.g. packaging, peer notification card and result report card design, sites coordination, personnel training, pilot study), consumables (SST kits, standard of care testing supplies), capital costs (office equipment), and personnel costs. The time horizon will be the duration of the trial.

### Statistical methods

#### Sample size

We used an inequality test to calculate the sample size (PASS 15.0, NCSS, LLC). The details of the sample size calculation are shown in Table 2. Preliminary study results from the pilot RCT showed that the number of alters by an index was 0.05 through standard of care delivery, 0.60 through standard SST delivery, and 0.45 through SST link delivery. Based on the above data, we assumed that the variances of the three groups were equal with the same standard deviation of 0.8. Assuming the number of clusters will be allocated 1:1:1 to each arm, with cluster size of 10 per cluster, alpha of 0.05, power of 0.8, inter class correlation of 0.01, lost to follow-up rate of 0.20, we will need a minimum of 10 clusters per arm. To integrate the sample size calculations and to consider practicality, the final sample size would be ten clusters in standard SST delivery arm, ten clusters in SST referral link delivery arm, and ten clusters in standard of care arm, with a total cluster size of 30 (300 participants).

**Table 2:** Sample size calculations

**SST and standard of care**	ICC	0.01
	Mean difference	0.55
	Standard deviation	0.8
	COV of cluster sizes	0.65
	Number of people per cluster	10
	Alpha	0.05
	Power	0.8
	Total sample size per arm	60
	Number of clusters per arm	6
**LINK and standard of care**	ICC	0.01
	Mean difference	0.4
	Standard deviation	0.8
	COV of cluster sizes	0.65
	Number of people per cluster	10
	Alpha	0.05
	Power	0.8
	Total sample size per arm	80
	Number of clusters per arm	8

*ICC* Intra-class correlation, *COV* Coefficient of variation

### Data analysis

#### Primary analysis

Sociodemographic characteristics will be summarized using descriptive statistics. The mean number of alters who returned photo verified syphilis testing result in each arm will be assessed using intention-to-treat analysis during the distribution period (3 months). The effects of the intervention will be measured by comparing the mean number of alters who returned photo-verified syphilis testing results in the three arms by using generalized estimated equations modelling to account for potential correlations in outcomes within groups. Egocentric network analysis will be used to describe the network characteristics, such as network size (i.e., the total number of alters in index’s disclosure network) [24].

#### Secondary analysis

Within each arm of the study, we will calculate the proportion of first-time testers and testers with a positive syphilis testing result. Differences in proportions of first-time testers and testers with a positive syphilis testing result among alters during distribution period will be explored using Chi-square test and logistic regression in a multivariable model. We will also use descriptive analysis and chi-square test to compare the incidence of adverse events in three arms.

#### Economic evaluation

We will estimate the cost of syphilis testing in all three study arms. Costs will be categorized as fixed or variable costs. Fixed cost refers to cost that is independent of the number of tests conducted, including cost of start-up (see above), building rent and office equipment. Variable cost refers to cost that is dependent on the number of tests conducted, including SST kits, standard of care testing supplies, and personnel cost. Personnel costs will be calculated by multiplying the staff time associated with each program activity by the compensation received by the staff who perform these activities. We will first calculate the total cost for each group, then divide these costs by the number of participants tested and by the number of cases diagnosed with syphilis. For each arm of the study, we will calculate the incremental cost per person tested and the incremental cost per person diagnosed. To identify the optimal strategy, we will rank the incremental cost-effectiveness ratios of the three arms. We will also examine uncertainty using univariate, multivariate and probabilistic sensitivity analyses from a decision tree model created used TreeAgePro 2020, R2 (TreeAge Software, Williamstown, MA).

### Missing data plan

We anticipate the loss to follow-up will be less than 20% in the 3-month follow up. If the primary outcome is missing for < 15% of participants, analyses will use a complete-case approach. If an outcome is missing for ≥15% of participants, missingness mechanism will be investigated and multiple imputation will be used if suitable.

## Discussion

The prevalence of syphilis has remained high among MSM living in China while testing uptake is relatively low. A recent study demonstrated that using syphilis self-testing could increase the testing among this population [10]. However, there is limited evidence for the implementation of syphilis self-testing distribution models. In this study, we will test three syphilis testing models through social network distribution in a cluster randomized controlled trial. With the successful experience of HIVST secondary distribution and effectiveness of SST, we hypothesize that social network distribution of syphilis self-testing could increase the testing uptake among MSM in China.

This study examines the effectiveness of social network distribution on syphilis self-testing among MSM in China. Different from past studies on social network distribution, alters in our study could become secondary indexes. Alters of secondary indexes can become the next indexes as they wish. Our strategy may also empower MSM communities in expanding syphilis testing by using their own social networks. Participant recruitment will take place in facilities (community-based organization and specialist clinics), which offers better linkage to care for participants.

Several challenges might emerge in the implementation of this study. First, adverse events such as forced testing, physical and verbal abuse among participants, being misunderstood during the delivery procedures may occur. The findings of these questions will provide insights to design better syphilis self-testing support. Second, existing intervention delivery cannot guarantee all three packages or links are distributed. To encourage distribution, indexes will receive 3 USD for each successful distribution. In addition, we will try to understand why indexes fail to distribute any or partial packages via our survey. This information will help to improve current distribution models. Third, we ask for a refundable deposit in the SST arm if indexes wish to apply for SST packages again or secondary indexes wish to apply for SST packages. This might greatly discourage participation. For future studies, we need to discuss whether or not we should keep using this refundable deposit or adjust the amount if recruitment needs to be expanded. Lastly, though we used an objective measure for our primary outcome (photo verified syphilis testing results), there is a possibility that we underestimate the number of alters testing for syphilis if they don’t upload their testing results. We try to mitigate this by offering alters monetary incentives after their testing result is verified by a research assistant.

There are some limitations in this study. First, recruitment takes places in specialist hospitals and local CBOs. This will exclude individuals who are less likely to attend these recruitment sites, leading to sample bias. Second, we use data from treponemal test results, which might overlook testers’ treatment history and clinical data that help differentiate new and old cases.

In conclusion, this study will provide data on optimizing syphilis self-testing programs to increase syphilis testing uptake and earlier syphilis diagnosis among MSM in China.

## Supplementary Information

**Additional file 1.**

## Data Availability

The datasets used and/or analyzed during the current study are available from the corresponding author on reasonable request.

## Abbreviations

CBO: Community-based organization
CEA: Cost-effectiveness analysis
cRCT: cluster randomized controlled trial
HIV: Human immunodeficiency viruses
HIVST: HIV self-testing
LGBT: Lesbian, gay, bisexual, and transgender
LMICs: Low- and middle-income countries
MSM: Men who have sex with men
POC: Point-of-Care
SST: Syphilis self-testing
STI: Sexually transmitted infection
VCT: Voluntary counselling and testing
WHO: World Health Organization.
