# Supplementary material for: Social network distribution of syphilis self-testing among men who have sex with men in China: study protocol for a cluster randomized control trial
Source: BMC Infect Dis. 2021 May 27;21:491. doi: 10.1186/s12879-021-06137-0 (PMC8161568; doi:10.1186/s12879-021-06137-0)
Supplement: Supplementary file 1 — Additional file 1. [file 12879_2021_6137_MOESM1_ESM.docx]

**Table 1 Baseline characteristics of index in three arms**

| Characteristics | Total | Link Delivery | SST delivery | Standard of care | P value |
| --- | --- | --- | --- | --- | --- |
| ***Number of indexes*** | 60 | 20 | 20 | 20 |  |
| ***Demographic*** |  |  |  |  |  |
| Age |  |  |  |  |  |
| Mean ± sd | 31.4±9.2 | 33.4±7.6 | 31.5±9.9 | 29.4±9.9 |  |
| Median (IQR) | 28.5(25-36) | 32.5(28-37.5) | 28(23-39) | 27(24.5-29) |  |
| Marital status |  |  |  |  | 1.000 |
| Married | 12(20.0) | 4(20.0) | 4(20.0) | 4(20.0) |  |
| Unmarried | 48(80.0) | 16(80.0) | 16(80.0) | 16(80.0) |  |
| Highest education |  |  |  |  | 0.535 |
| High school or below | 24(40.0) | 9(45.0) | 6(30.0) | 9(45.0) |  |
| College or above | 36(60.0) | 11(55.0) | 14(70.0) | 11(55.0) |  |
| Monthly income |  |  |  |  | 0.098 |
| <=3000 | 8(13.3) | 0(0.0) | 5(25.0) | 3(15.0) |  |
| 3001-8000 | 44(73.3) | 16(80.0) | 12(60.0) | 16(80.0) |  |
| >8000 | 8(13.3) | 4(20.0) | 3(15.0) | 1(5.0) |  |
| ***Sexual behavior*** |  |  |  |  |  |
| Had anal sex with men in the past month | 50(83.3) | 15(75.0) | 19(95.0) | 16(80.0) | 0.305 |
| Number of male partners in the past month (median, IQR) | 2(1-3) | 2(1-3) | 2(1-3) | 1(1-3) |  |
| Consistent condom use of anal sex in the past month | 24(48.0) | 7(46.7) | 9(47.4) | 8(50.0) | 0.981 |
| Condom use in the past anal sex | 42(84.0) | 13(86.7) | 15(79.0) | 14(87.5) | 0.795 |
| Number of male regular partner in the past month (median, IQR) | 2(1-2) | 2(0-2) | 2(1-2) | 2(1-2) |  |
| Consistent condom use of anal sex with regular partner in the past month | 18(48.7) | 6(50.0) | 5(45.5) | 7(50.0) | 0.969 |
| Number of male casual partner in the past month (median, IQR) | 1(1-2) | 1.5(0-2) | 1(1-2) | 1.5(1-2) |  |
| Consistent condom use of anal sex with casual partner in the past month | 17(58.6) | 6(60.0) | 5(55.6) | 6(60.0) | 1.000 |
| Had group sex with men in the past month | 8(16.0) | 2(13.3) | 4(21.1) | 2(12.5) | 0.795 |
| Had sex with women in the past month | 7(11.7) | 3(15.0) | 3(15.0) | 1(5.0) | 0.680 |
| Drug use before or during sex in the past month | 16(26.7) | 7(35.0) | 4(20.0) | 5(25.0) | 0.551 |
| ***Testing behavior*** |  |  |  |  |  |
| Ever had syphilis testing | 46(76.7) | 18(90.0) | 15(75.0) | 13(65.0) | 0.207 |
| Ever had syphilis self-testing | 22(47.8) | 8(44.4) | 7(46.7) | 7(53.9) | 0.870 |
| Ever had HIV testing | 53(88.3) | 18(90.0) | 17(85.0) | 18(90.0) | 1.000 |
| Ever had HIV self-testing | 33(62.3) | 9(50.0) | 11(64.7) | 13(72.2) | 0.376 |
| Ever had other STIs testing | 6(10.0) | 5(25.0) | 0(0.0) | 1(5.0) | **0.040** |

**Table 2 Motivated syphilis testing uptake in three arms**

| Arms | Mean number of motivated syphilis testers | Numbers of clusters | Mean difference | 95% CI | P value |
| --- | --- | --- | --- | --- | --- |
| Link Delivery | 0.45 (0.97) | 2 | 0.40 | -0.04-0.84 | 0.073 |
| SST delivery | 0.60 (1.07) | 2 | 0.55 | **0.07-1.03** | **0.024** |
| Standard of care | 0.05 (0.22) | 2 | NA | NA | NA |

Data given as mean (standard deviation). NA=not applicable.
